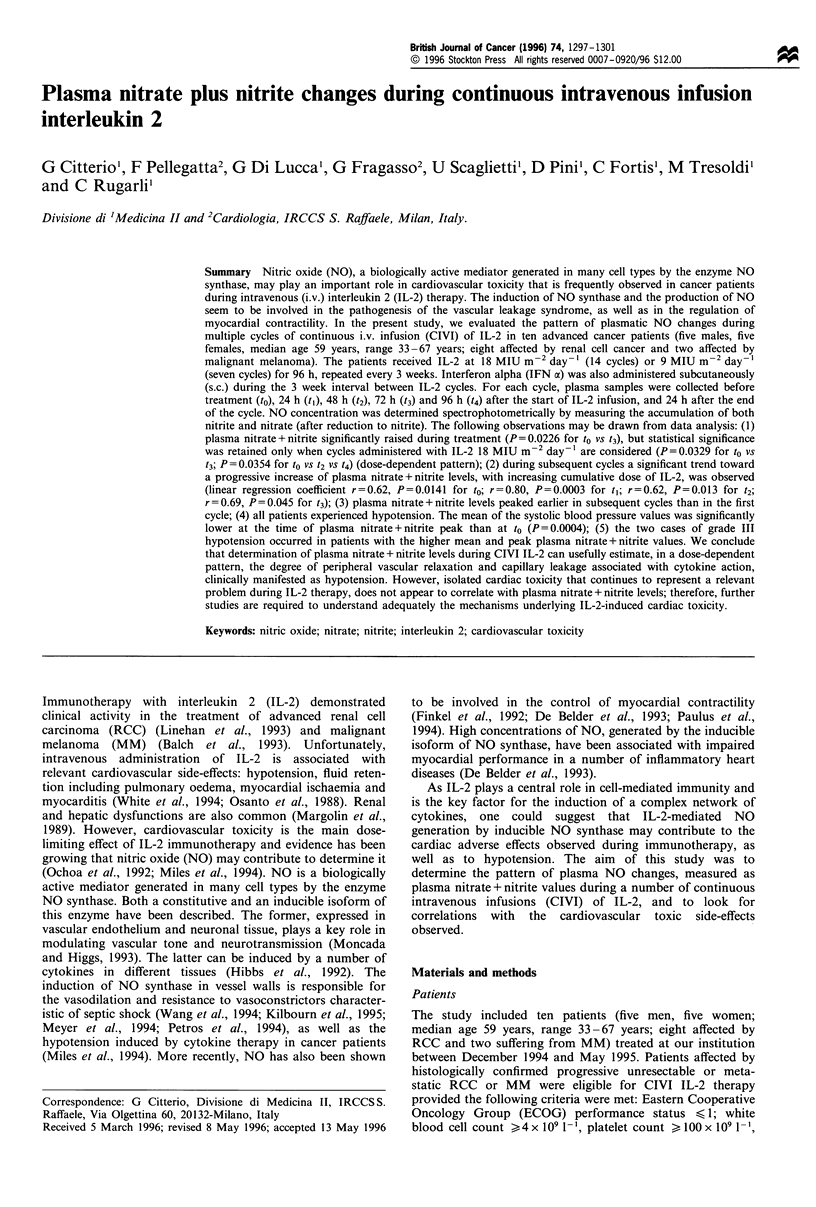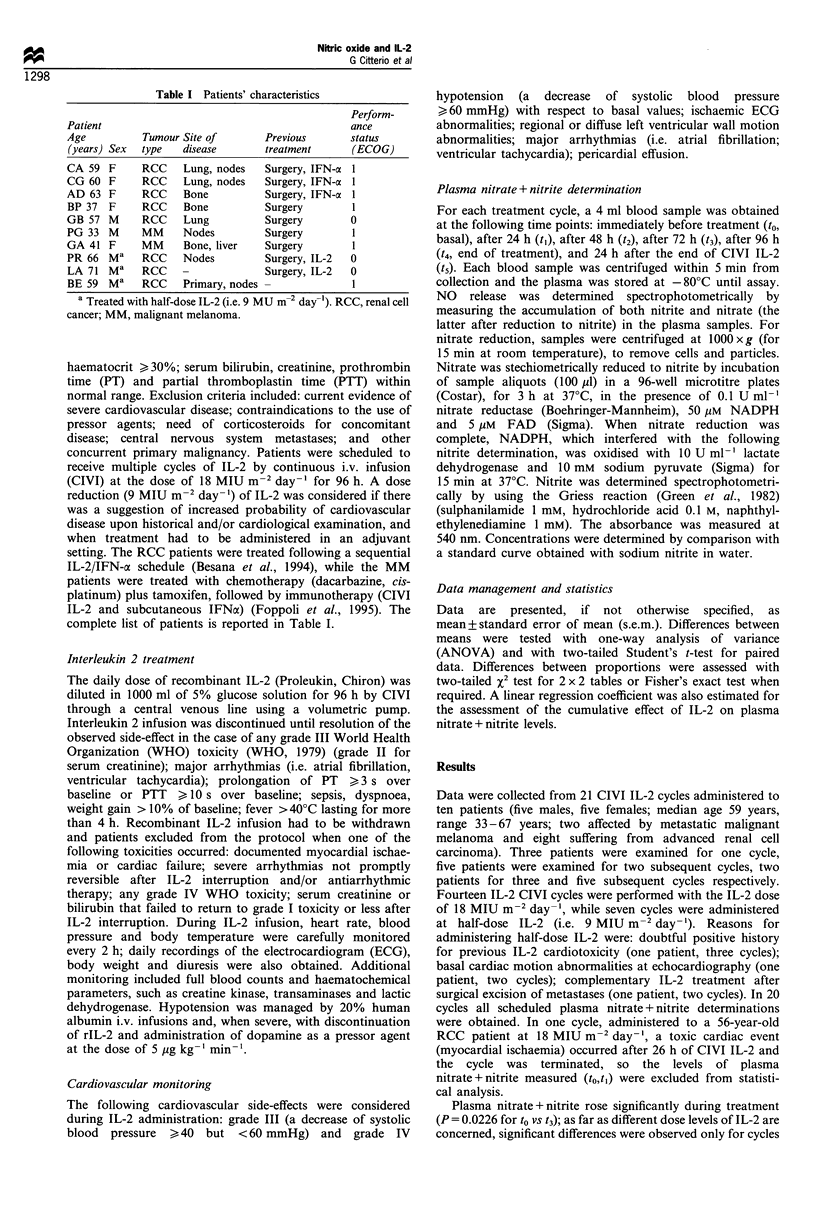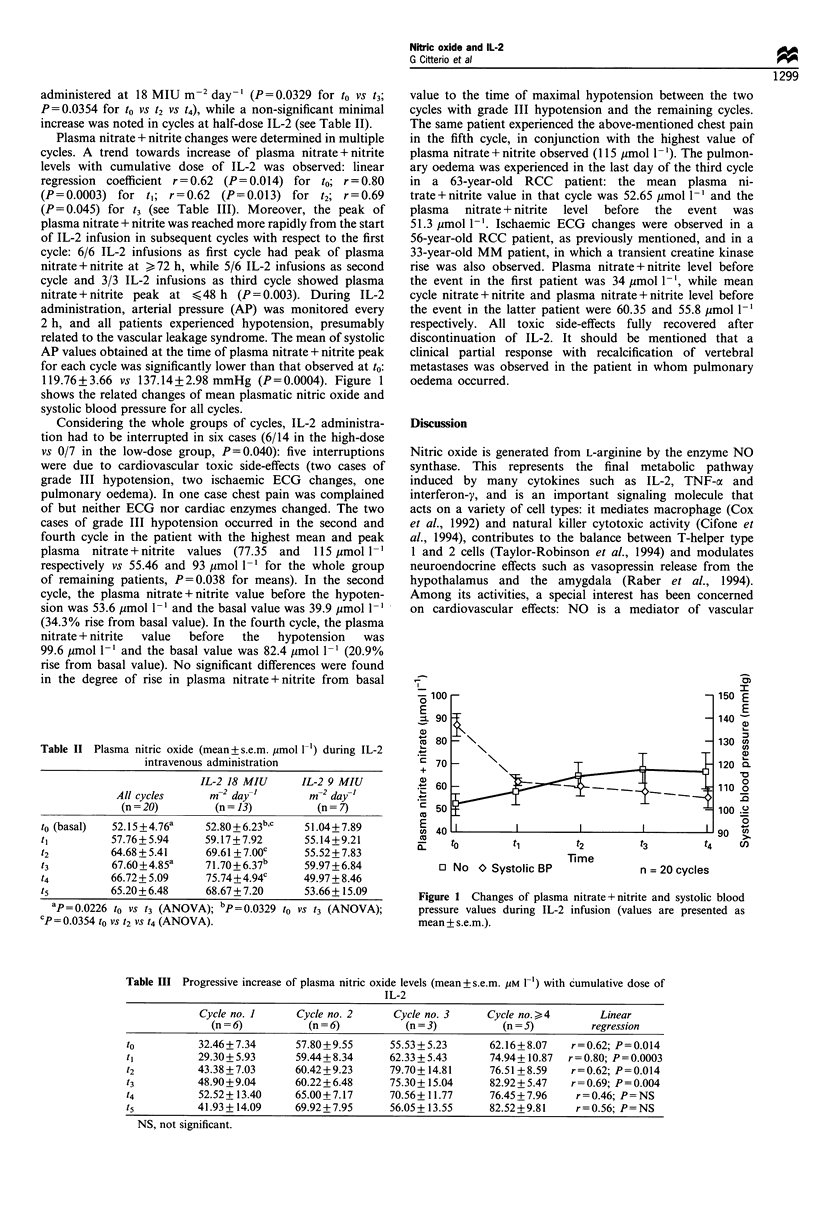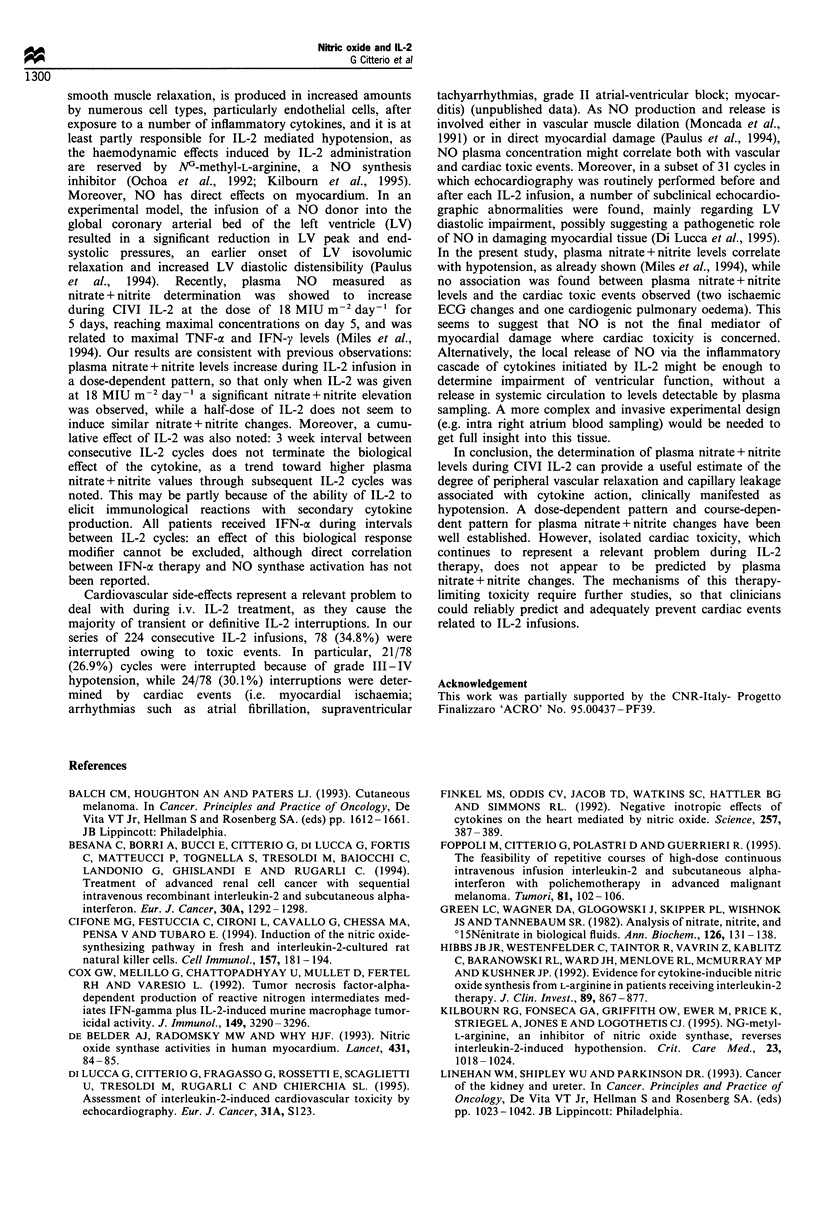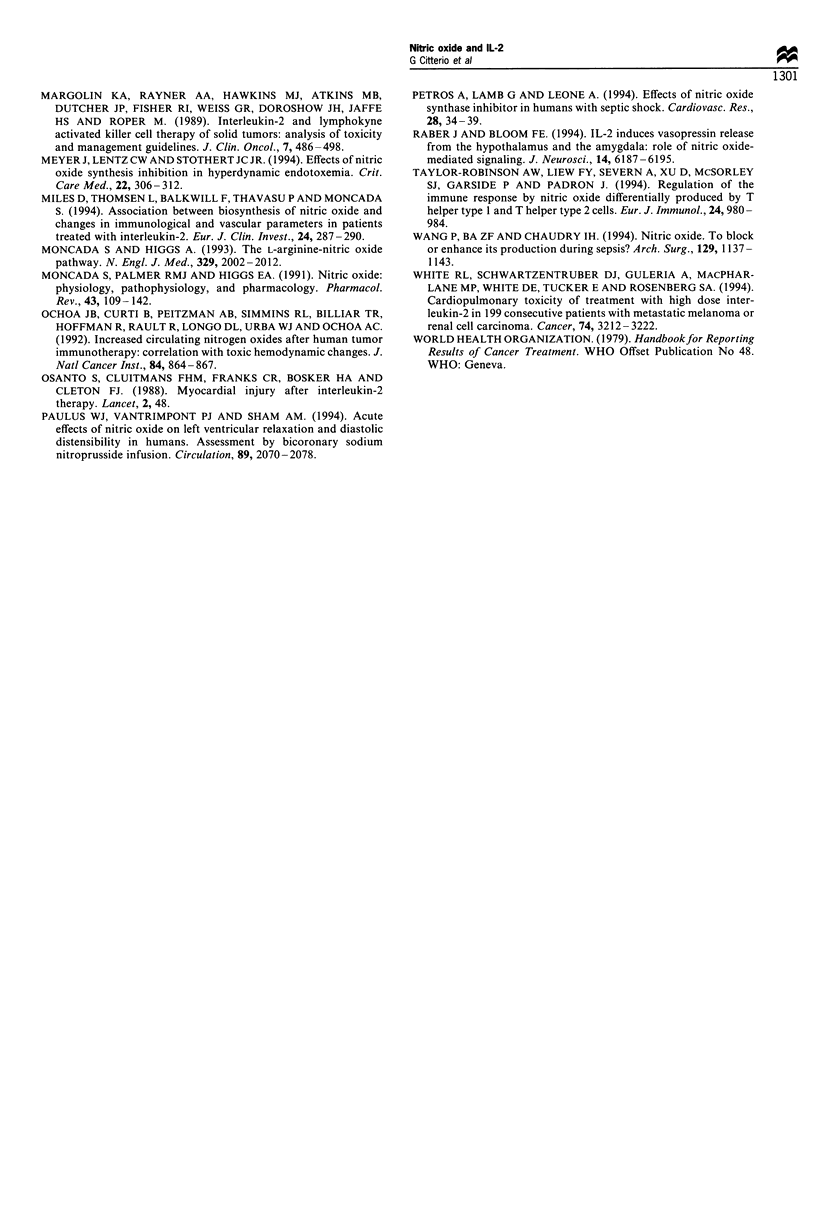# Plasma nitrate plus nitrite changes during continuous intravenous infusion interleukin 2.

**DOI:** 10.1038/bjc.1996.533

**Published:** 1996-10

**Authors:** G. Citterio, F. Pellegatta, G. D. Lucca, G. Fragasso, U. Scaglietti, D. Pini, C. Fortis, M. Tresoldi, C. Rugarli

**Affiliations:** Divisione di Medicina II, IRCCS S. Raffaele, Milan, Italy.

## Abstract

Nitric oxide (NO), a biologically active mediator generated in many cell types by the enzyme NO synthase, may play an important role in cardiovascular toxicity that is frequently observed in cancer patients during intravenous (i.v.) interleukin 2 (IL-2) therapy. The induction of NO synthase and the production of NO seem to be involved in the pathogenesis of the vascular leakage syndrome, as well as in the regulation of myocardial contractility. In the present study, we evaluated the pattern of plasmatic NO changes during multiple cycles of continuous i.v. infusion (CIVI) of IL-2 in ten advanced cancer patients (five males, five females, median age 59 years, range 33-67 years; eight affected by renal cell cancer and two affected by malignant melanoma). The patients received IL-2 at 18 MIU m-2 day-1 (14 cycles) or 9 MIU m-2 day-1 (seven cycles) for 96 h, repeated every 3 weeks. Interferon alpha (IFN alpha) was also administered subcutaneously (s.c) during the 3 week interval between IL-2 cycles. For each cycle, plasma samples were collected before treatment (t0), 24 h (t1), 48 h (t2), 72 h (t3) and 96 h (t4) after the start of IL-2 infusion, and 24 h after the end of the cycle. NO concentration was determined spectrophotometrically by measuring the accumulation of both nitrite and nitrate (after reduction to nitrite). The following observations may be drawn from data analysis: (1) plasma nitrate + nitrite significantly raised during treatment (P = 0.0226 for t0 vs t3), but statistical significance was retained only when cycles administered with IL-2 18 MIU m-2 day-1 are considered (P = 0.0329 for t0 vs t3; P = 0.0354 for t0 vs t2 vs t4) (dose-dependent pattern); (2) during subsequent cycles a significant trend toward a progressive increase of plasma nitrate + nitrite levels, with increasing cumulative dose of IL-2, was observed (linear regression coefficient r = 0.62, P = 0.0141 for t0; r = 0.80, P = 0.0003 for t1; r = 0.62, P = 0.013 for t2; r = 0.69, P = 0.045 for t3); (3) plasma nitrate + nitrite levels peaked earlier in subsequent cycles than in the first cycle; (4) all patients experienced hypotension. The mean of the systolic blood pressure values was significantly lower at the time of plasma nitrate + nitrite peak than at t0 (P = 0.0004); (5) the two cases of grade III hypotension occurred in patients with the higher mean and peak plasma nitrate + nitrite values. We conclude that determination of plasma nitrate + nitrite levels during CIVI IL-2 can usefully estimate, in a dose-dependent pattern, the degree of peripheral vascular relaxation and capillary leakage associated with cytokine action, clinically manifested as hypotension. However, isolated cardiac toxicity that continues to represent a relevant problem during IL-2 therapy, does not appear to correlate with plasma nitrate + nitrite levels; therefore, further studies are required to understand adequately the mechanisms underlying IL-2-induced cardiac toxicity.


British Journal of Cancer (1996) 74, 1297–1301
© 1996 Stockton Press All rights reserved 0007-0920/96 $12.00

### Plasma nitrate plus nitrite changes during continuous intravenous infusion interleukin 2

G Citterio[1], F Pellegatta[2], G Di Lucca[1], G Fragasso[2], U Scaglietti[1], D Pini[1], C Fortis[1], M Tresoldi[1] and C Rugarli[1]

*Divisione di [1]Medicina II and [2]Cardiologia, IRCCS S. Raffaele, Milan, Italy.*

**Summary** Nitric oxide (NO), a biologically active mediator generated in many cell types by the enzyme NO synthase, may play an important role in cardiovascular toxicity that is frequently observed in cancer patients during intravenous (i.v.) interleukin 2 (IL-2) therapy. The induction of NO synthase and the production of NO seem to be involved in the pathogenesis of the vascular leakage syndrome, as well as in the regulation of myocardial contractility. In the present study, we evaluated the pattern of plasmatic NO changes during multiple cycles of continuous i.v. infusion (CIVI) of IL-2 in ten advanced cancer patients (five males, five females, median age 59 years, range 33–67 years; eight affected by renal cell cancer and two affected by malignant melanoma). The patients received IL-2 at 18 MIU $m^{-2}$ $day^{-1}$ (14 cycles) or 9 MIU $m^{-2}$ $day^{-1}$ (seven cycles) for 96 h, repeated every 3 weeks. Interferon alpha (IFN α) was also administered subcutaneously (s.c.) during the 3 week interval between IL-2 cycles. For each cycle, plasma samples were collected before treatment ($t_0$), 24 h ($t_1$), 48 h ($t_2$), 72 h ($t_3$) and 96 h ($t_4$) after the start of IL-2 infusion, and 24 h after the end of the cycle. NO concentration was determined spectrophotometrically by measuring the accumulation of both nitrite and nitrate (after reduction to nitrite). The following observations may be drawn from data analysis: (1) plasma nitrate + nitrite significantly raised during treatment ($P$=0.0226 for $t_0$ *vs* $t_3$), but statistical significance was retained only when cycles administered with IL-2 18 MIU $m^{-2}$ $day^{-1}$ are considered ($P$=0.0329 for $t_0$ *vs* $t_3$; $P$=0.0354 for $t_0$ *vs* $t_2$ *vs* $t_4$) (dose-dependent pattern); (2) during subsequent cycles a significant trend toward a progressive increase of plasma nitrate + nitrite levels, with increasing cumulative dose of IL-2, was observed (linear regression coefficient $r$=0.62, $P$=0.0141 for $t_0$; $r$=0.80, $P$=0.0003 for $t_1$; $r$=0.62, $P$=0.013 for $t_2$; $r$=0.69, $P$=0.045 for $t_3$); (3) plasma nitrate + nitrite levels peaked earlier in subsequent cycles than in the first cycle; (4) all patients experienced hypotension. The mean of the systolic blood pressure values was significantly lower at the time of plasma nitrate + nitrite peak than at $t_0$ ($P$=0.0004); (5) the two cases of grade III hypotension occurred in patients with the higher mean and peak plasma nitrate + nitrite values. We conclude that determination of plasma nitrate + nitrite levels during CIVI IL-2 can usefully estimate, in a dose-dependent pattern, the degree of peripheral vascular relaxation and capillary leakage associated with cytokine action, clinically manifested as hypotension. However, isolated cardiac toxicity that continues to represent a relevant problem during IL-2 therapy, does not appear to correlate with plasma nitrate + nitrite levels; therefore, further studies are required to understand adequately the mechanisms underlying IL-2-induced cardiac toxicity.

**Keywords:** nitric oxide; nitrate; nitrite; interleukin 2; cardiovascular toxicity

Immunotherapy with interleukin 2 (IL-2) demonstrated clinical activity in the treatment of advanced renal cell carcinoma (RCC) (Linehan *et al.*, 1993) and malignant melanoma (MM) (Balch *et al.*, 1993). Unfortunately, intravenous administration of IL-2 is associated with relevant cardiovascular side-effects: hypotension, fluid retention including pulmonary oedema, myocardial ischaemia and myocarditis (White *et al.*, 1994; Osanto *et al.*, 1988). Renal and hepatic dysfunctions are also common (Margolin *et al.*, 1989). However, cardiovascular toxicity is the main dose-limiting effect of IL-2 immunotherapy and evidence has been growing that nitric oxide (NO) may contribute to determine it (Ochoa *et al.*, 1992; Miles *et al.*, 1994). NO is a biologically active mediator generated in many cell types by the enzyme NO synthase. Both a constitutive and an inducible isoform of this enzyme have been described. The former, expressed in vascular endothelium and neuronal tissue, plays a key role in modulating vascular tone and neurotransmission (Moncada and Higgs, 1993). The latter can be induced by a number of cytokines in different tissues (Hibbs *et al.*, 1992). The induction of NO synthase in vessel walls is responsible for the vasodilation and resistance to vasoconstrictors characteristic of septic shock (Wang *et al.*, 1994; Kilbourn *et al.*, 1995; Meyer *et al.*, 1994; Petros *et al.*, 1994), as well as the hypotension induced by cytokine therapy in cancer patients (Miles *et al.*, 1994). More recently, NO has also been shown to be involved in the control of myocardial contractility (Finkel *et al.*, 1992; De Belder *et al.*, 1993; Paulus *et al.*, 1994). High concentrations of NO, generated by the inducible isoform of NO synthase, have been associated with impaired myocardial performance in a number of inflammatory heart diseases (De Belder *et al.*, 1993).

As IL-2 plays a central role in cell-mediated immunity and is the key factor for the induction of a complex network of cytokines, one could suggest that IL-2-mediated NO generation by inducible NO synthase may contribute to the cardiac adverse effects observed during immunotherapy, as well as to hypotension. The aim of this study was to determine the pattern of plasma NO changes, measured as plasma nitrate + nitrite values during a number of continuous intravenous infusions (CIVI) of IL-2, and to look for correlations with the cardiovascular toxic side-effects observed.

### Materials and methods

### *Patients*

The study included ten patients (five men, five women; median age 59 years, range 33–67 years; eight affected by RCC and two suffering from MM) treated at our institution between December 1994 and May 1995. Patients affected by histologically confirmed progressive unresectable or metastatic RCC or MM were eligible for CIVI IL-2 therapy provided the following criteria were met: Eastern Cooperative Oncology Group (ECOG) performance status $\leqslant 1$; white blood cell count $\geqslant 4 \times 10^9\ l^{-1}$, platelet count $\geqslant 100 \times 10^9\ l^{-1}$,

Correspondence: G Citterio, Divisione di Medicina II, IRCCS S. Raffaele, Via Olgettina 60, 20132-Milano, Italy
Received 5 March 1996; revised 8 May 1996; accepted 13 May 1996

Table I Patients' characteristics

| Patient Age (years) | Sex | Tumour type | Site of disease | Previous treatment | Performance status (ECOG) |
|---|---|---|---|---|---|
| CA 59 | F | RCC | Lung, nodes | Surgery, IFN-α | 1 |
| CG 60 | F | RCC | Lung, nodes | Surgery, IFN-α | 1 |
| AD 63 | F | RCC | Bone | Surgery, IFN-α | 1 |
| BP 37 | F | RCC | Bone | Surgery | 1 |
| GB 57 | M | RCC | Lung | Surgery | 0 |
| PG 33 | M | MM | Nodes | Surgery | 1 |
| GA 41 | F | MM | Bone, liver | Surgery | 1 |
| PR 66 | M[a] | RCC | Nodes | Surgery, IL-2 | 0 |
| LA 71 | M[a] | RCC | – | Surgery, IL-2 | 0 |
| BE 59 | M[a] | RCC | Primary, nodes | – | 1 |

[a] Treated with half-dose IL-2 (i.e. 9 MU $m^{-2}$ $day^{-1}$). RCC, renal cell cancer; MM, malignant melanoma.

haematocrit ⩾30%; serum bilirubin, creatinine, prothrombin time (PT) and partial thromboplastin time (PTT) within normal range. Exclusion criteria included: current evidence of severe cardiovascular disease; contraindications to the use of pressor agents; need of corticosteroids for concomitant disease; central nervous system metastases; and other concurrent primary malignancy. Patients were scheduled to receive multiple cycles of IL-2 by continuous i.v. infusion (CIVI) at the dose of 18 MIU $m^{-2}$ $day^{-1}$ for 96 h. A dose reduction (9 MIU $m^{-2}$ $day^{-1}$) of IL-2 was considered if there was a suggestion of increased probability of cardiovascular disease upon historical and/or cardiological examination, and when treatment had to be administered in an adjuvant setting. The RCC patients were treated following a sequential IL-2/IFN-α schedule (Besana *et al.*, 1994), while the MM patients were treated with chemotherapy (dacarbazine, *cis*-platinum) plus tamoxifen, followed by immunotherapy (CIVI IL-2 and subcutaneous IFNα) (Foppoli *et al.*, 1995). The complete list of patients is reported in Table I.

*Interleukin 2 treatment*

The daily dose of recombinant IL-2 (Proleukin, Chiron) was diluted in 1000 ml of 5% glucose solution for 96 h by CIVI through a central venous line using a volumetric pump. Interleukin 2 infusion was discontinued until resolution of the observed side-effect in the case of any grade III World Health Organization (WHO) toxicity (WHO, 1979) (grade II for serum creatinine); major arrhythmias (i.e. atrial fibrillation, ventricular tachycardia); prolongation of PT ⩾3 s over baseline or PTT ⩾10 s over baseline; sepsis, dyspnoea, weight gain >10% of baseline; fever >40°C lasting for more than 4 h. Recombinant IL-2 infusion had to be withdrawn and patients excluded from the protocol when one of the following toxicities occurred: documented myocardial ischaemia or cardiac failure; severe arrhythmias not promptly reversible after IL-2 interruption and/or antiarrhythmic therapy; any grade IV WHO toxicity; serum creatinine or bilirubin that failed to return to grade I toxicity or less after IL-2 interruption. During IL-2 infusion, heart rate, blood pressure and body temperature were carefully monitored every 2 h; daily recordings of the electrocardiogram (ECG), body weight and diuresis were also obtained. Additional monitoring included full blood counts and haematochemical parameters, such as creatine kinase, transaminases and lactic dehydrogenase. Hypotension was managed by 20% human albumin i.v. infusions and, when severe, with discontinuation of rIL-2 and administration of dopamine as a pressor agent at the dose of 5 $\mu$g $kg^{-1}$ $min^{-1}$.

*Cardiovascular monitoring*

The following cardiovascular side-effects were considered during IL-2 administration: grade III (a decrease of systolic blood pressure ⩾40 but <60 mmHg) and grade IV hypotension (a decrease of systolic blood pressure ⩾60 mmHg) with respect to basal values; ischaemic ECG abnormalities; regional or diffuse left ventricular wall motion abnormalities; major arrhythmias (i.e. atrial fibrillation; ventricular tachycardia); pericardial effusion.

*Plasma nitrate + nitrite determination*

For each treatment cycle, a 4 ml blood sample was obtained at the following time points: immediately before treatment ($t_0$, basal), after 24 h ($t_1$), after 48 h ($t_2$), after 72 h ($t_3$), after 96 h ($t_4$, end of treatment), and 24 h after the end of CIVI IL-2 ($t_5$). Each blood sample was centrifuged within 5 min from collection and the plasma was stored at −80°C until assay. NO release was determined spectrophotometrically by measuring the accumulation of both nitrite and nitrate (the latter after reduction to nitrite) in the plasma samples. For nitrate reduction, samples were centrifuged at 1000 × *g* (for 15 min at room temperature), to remove cells and particles. Nitrate was stechiometrically reduced to nitrite by incubation of sample aliquots (100 $\mu$l) in a 96-well microtitre plates (Costar), for 3 h at 37°C, in the presence of 0.1 U $ml^{-1}$ nitrate reductase (Boehringer-Mannheim), 50 $\mu$M NADPH and 5 $\mu$M FAD (Sigma). When nitrate reduction was complete, NADPH, which interfered with the following nitrite determination, was oxidised with 10 U $ml^{-1}$ lactate dehydrogenase and 10 mM sodium pyruvate (Sigma) for 15 min at 37°C. Nitrite was determined spectrophotometrically by using the Griess reaction (Green *et al.*, 1982) (sulphanilamide 1 mM, hydrochloride acid 0.1 M, naphthylethylenediamine 1 mM). The absorbance was measured at 540 nm. Concentrations were determined by comparison with a standard curve obtained with sodium nitrite in water.

*Data management and statistics*

Data are presented, if not otherwise specified, as mean ± standard error of mean (s.e.m.). Differences between means were tested with one-way analysis of variance (ANOVA) and with two-tailed Student's *t*-test for paired data. Differences between proportions were assessed with two-tailed $\chi^2$ test for 2 × 2 tables or Fisher's exact test when required. A linear regression coefficient was also estimated for the assessment of the cumulative effect of IL-2 on plasma nitrate + nitrite levels.

### Results

Data were collected from 21 CIVI IL-2 cycles administered to ten patients (five males, five females; median age 59 years, range 33–67 years; two affected by metastatic malignant melanoma and eight suffering from advanced renal cell carcinoma). Three patients were examined for one cycle, five patients were examined for two subsequent cycles, two patients for three and five subsequent cycles respectively. Fourteen IL-2 CIVI cycles were performed with the IL-2 dose of 18 MIU $m^{-2}$ $day^{-1}$, while seven cycles were administered at half-dose IL-2 (i.e. 9 MIU $m^{-2}$ $day^{-1}$). Reasons for administering half-dose IL-2 were: doubtful positive history for previous IL-2 cardiotoxicity (one patient, three cycles); basal cardiac motion abnormalities at echocardiography (one patient, two cycles); complementary IL-2 treatment after surgical excision of metastases (one patient, two cycles). In 20 cycles all scheduled plasma nitrate + nitrite determinations were obtained. In one cycle, administered to a 56-year-old RCC patient at 18 MIU $m^{-2}$ $day^{-1}$, a toxic cardiac event (myocardial ischaemia) occurred after 26 h of CIVI IL-2 and the cycle was terminated, so the levels of plasma nitrate + nitrite measured ($t_0$,$t_1$) were excluded from statistical analysis.

Plasma nitrate + nitrite rose significantly during treatment ($P = 0.0226$ for $t_0$ *vs* $t_3$); as far as different dose levels of IL-2 are concerned, significant differences were observed only for cycles administered at 18 MIU $m^{-2}$ $day^{-1}$ ($P=0.0329$ for $t_0$ *vs* $t_3$; $P=0.0354$ for $t_0$ *vs* $t_2$ *vs* $t_4$), while a non-significant minimal increase was noted in cycles at half-dose IL-2 (see Table II).

Plasma nitrate + nitrite changes were determined in multiple cycles. A trend towards increase of plasma nitrate + nitrite levels with cumulative dose of IL-2 was observed: linear regression coefficient $r=0.62$ ($P=0.014$) for $t_0$; $r=0.80$ ($P=0.0003$) for $t_1$; $r=0.62$ ($P=0.013$) for $t_2$; $r=0.69$ ($P=0.045$) for $t_3$ (see Table III). Moreover, the peak of plasma nitrate + nitrite was reached more rapidly from the start of IL-2 infusion in subsequent cycles with respect to the first cycle: 6/6 IL-2 infusions as first cycle had peak of plasma nitrate + nitrite at $\geqslant$72 h, while 5/6 IL-2 infusions as second cycle and 3/3 IL-2 infusions as third cycle showed plasma nitrate + nitrite peak at $\leqslant$48 h ($P=0.003$). During IL-2 administration, arterial pressure (AP) was monitored every 2 h, and all patients experienced hypotension, presumably related to the vascular leakage syndrome. The mean of systolic AP values obtained at the time of plasma nitrate + nitrite peak for each cycle was significantly lower than that observed at $t_0$: $119.76\pm3.66$ *vs* $137.14\pm2.98$ mmHg ($P=0.0004$). Figure 1 shows the related changes of mean plasmatic nitric oxide and systolic blood pressure for all cycles.

Considering the whole groups of cycles, IL-2 administration had to be interrupted in six cases (6/14 in the high-dose *vs* 0/7 in the low-dose group, $P=0.040$): five interruptions were due to cardiovascular toxic side-effects (two cases of grade III hypotension, two ischaemic ECG changes, one pulmonary oedema). In one case chest pain was complained of but neither ECG nor cardiac enzymes changed. The two cases of grade III hypotension occurred in the second and fourth cycle in the patient with the highest mean and peak plasma nitrate + nitrite values (77.35 and 115 $\mu$mol $l^{-1}$ respectively *vs* 55.46 and 93 $\mu$mol $l^{-1}$ for the whole group of remaining patients, $P=0.038$ for means). In the second cycle, the plasma nitrate + nitrite value before the hypotension was 53.6 $\mu$mol $l^{-1}$ and the basal value was 39.9 $\mu$mol $l^{-1}$ (34.3% rise from basal value). In the fourth cycle, the plasma nitrate + nitrite value before the hypotension was 99.6 $\mu$mol $l^{-1}$ and the basal value was 82.4 $\mu$mol $l^{-1}$ (20.9% rise from basal value). No significant differences were found in the degree of rise in plasma nitrate + nitrite from basal value to the time of maximal hypotension between the two cycles with grade III hypotension and the remaining cycles. The same patient experienced the above-mentioned chest pain in the fifth cycle, in conjunction with the highest value of plasma nitrate + nitrite observed (115 $\mu$mol $l^{-1}$). The pulmonary oedema was experienced in the last day of the third cycle in a 63-year-old RCC patient: the mean plasma nitrate + nitrite value in that cycle was 52.65 $\mu$mol $l^{-1}$ and the plasma nitrate + nitrite level before the event was 51.3 $\mu$mol $l^{-1}$. Ischaemic ECG changes were observed in a 56-year-old RCC patient, as previously mentioned, and in a 33-year-old MM patient, in which a transient creatine kinase rise was also observed. Plasma nitrate + nitrite level before the event in the first patient was 34 $\mu$mol $l^{-1}$, while mean cycle nitrate + nitrite and plasma nitrate + nitrite level before the event in the latter patient were 60.35 and 55.8 $\mu$mol $l^{-1}$ respectively. All toxic side-effects fully recovered after discontinuation of IL-2. It should be mentioned that a clinical partial response with recalcification of vertebral metastases was observed in the patient in whom pulmonary oedema occurred.

### Discussion

Nitric oxide is generated from L-arginine by the enzyme NO synthase. This represents the final metabolic pathway induced by many cytokines such as IL-2, TNF-$\alpha$ and interferon-$\gamma$, and is an important signaling molecule that acts on a variety of cell types: it mediates macrophage (Cox *et al.*, 1992) and natural killer cytotoxic activity (Cifone *et al.*, 1994), contributes to the balance between T-helper type 1 and 2 cells (Taylor-Robinson *et al.*, 1994) and modulates neuroendocrine effects such as vasopressin release from the hypothalamus and the amygdala (Raber *et al.*, 1994). Among its activities, a special interest has been concerned on cardiovascular effects: NO is a mediator of vascular

**Table II** Plasma nitric oxide (mean $\pm$ s.e.m. $\mu$mol $l^{-1}$) during IL-2 intravenous administration

| | *All cycles* (n = *20*) | *IL-2 18 MIU* $m^{-2}$ $day^{-1}$ (n = *13*) | *IL-2 9 MIU* $m^{-2}$ $day^{-1}$ (n = *7*) |
|---|---|---|---|
| $t_0$ (basal) | $52.15\pm4.76^a$ | $52.80\pm6.23^{b,c}$ | $51.04\pm7.89$ |
| $t_1$ | $57.76\pm5.94$ | $59.17\pm7.92$ | $55.14\pm9.21$ |
| $t_2$ | $64.68\pm5.41$ | $69.61\pm7.00^c$ | $55.52\pm7.83$ |
| $t_3$ | $67.60\pm4.85^a$ | $71.70\pm6.37^b$ | $59.97\pm6.84$ |
| $t_4$ | $66.72\pm5.09$ | $75.74\pm4.94^c$ | $49.97\pm8.46$ |
| $t_5$ | $65.20\pm6.48$ | $68.67\pm7.20$ | $53.66\pm15.09$ |

[a]$P=0.0226$ $t_0$ *vs* $t_3$ (ANOVA); [b]$P=0.0329$ $t_0$ *vs* $t_3$ (ANOVA); [c]$P=0.0354$ $t_0$ *vs* $t_2$ *vs* $t_4$ (ANOVA).

**Figure 1** Changes of plasma nitrate + nitrite and systolic blood pressure values during IL-2 infusion (values are presented as mean $\pm$ s.e.m.).

**Table III** Progressive increase of plasma nitric oxide levels (mean $\pm$ s.e.m. $\mu$M $l^{-1}$) with cumulative dose of IL-2

| | *Cycle no. 1* (n = *6*) | *Cycle no. 2* (n = *6*) | *Cycle no. 3* (n = *3*) | *Cycle no.* $\geqslant$4 (n = *5*) | *Linear regression* |
|---|---|---|---|---|---|
| $t_0$ | $32.46\pm7.34$ | $57.80\pm9.55$ | $55.53\pm5.23$ | $62.16\pm8.07$ | $r=0.62$; $P=0.014$ |
| $t_1$ | $29.30\pm5.93$ | $59.44\pm8.34$ | $62.33\pm5.43$ | $74.94\pm10.87$ | $r=0.80$; $P=0.0003$ |
| $t_2$ | $43.38\pm7.03$ | $60.42\pm9.23$ | $79.70\pm14.81$ | $76.51\pm8.59$ | $r=0.62$; $P=0.014$ |
| $t_3$ | $48.90\pm9.04$ | $60.22\pm6.48$ | $75.30\pm15.04$ | $82.92\pm5.47$ | $r=0.69$; $P=0.004$ |
| $t_4$ | $52.52\pm13.40$ | $65.00\pm7.17$ | $70.56\pm11.77$ | $76.45\pm7.96$ | $r=0.46$; $P=$NS |
| $t_5$ | $41.93\pm14.09$ | $69.92\pm7.95$ | $56.05\pm13.55$ | $82.52\pm9.81$ | $r=0.56$; $P=$NS |

NS, not significant.

smooth muscle relaxation, is produced in increased amounts by numerous cell types, particularly endothelial cells, after exposure to a number of inflammatory cytokines, and it is at least partly responsible for IL-2 mediated hypotension, as the haemodynamic effects induced by IL-2 administration are reserved by $N^G$-methyl-L-arginine, a NO synthesis inhibitor (Ochoa *et al.*, 1992; Kilbourn *et al.*, 1995). Moreover, NO has direct effects on myocardium. In an experimental model, the infusion of a NO donor into the global coronary arterial bed of the left ventricle (LV) resulted in a significant reduction in LV peak and end-systolic pressures, an earlier onset of LV isovolumic relaxation and increased LV diastolic distensibility (Paulus *et al.*, 1994). Recently, plasma NO measured as nitrate + nitrite determination was showed to increase during CIVI IL-2 at the dose of 18 MIU $m^{-2}$ $day^{-1}$ for 5 days, reaching maximal concentrations on day 5, and was related to maximal TNF-$\alpha$ and IFN-$\gamma$ levels (Miles *et al.*, 1994). Our results are consistent with previous observations: plasma nitrate + nitrite levels increase during IL-2 infusion in a dose-dependent pattern, so that only when IL-2 was given at 18 MIU $m^{-2}$ $day^{-1}$ a significant nitrate + nitrite elevation was observed, while a half-dose of IL-2 does not seem to induce similar nitrate + nitrite changes. Moreover, a cumulative effect of IL-2 was also noted: 3 week interval between consecutive IL-2 cycles does not terminate the biological effect of the cytokine, as a trend toward higher plasma nitrate + nitrite values through subsequent IL-2 cycles was noted. This may be partly because of the ability of IL-2 to elicit immunological reactions with secondary cytokine production. All patients received IFN-$\alpha$ during intervals between IL-2 cycles: an effect of this biological response modifier cannot be excluded, although direct correlation between IFN-$\alpha$ therapy and NO synthase activation has not been reported.

Cardiovascular side-effects represent a relevant problem to deal with during i.v. IL-2 treatment, as they cause the majority of transient or definitive IL-2 interruptions. In our series of 224 consecutive IL-2 infusions, 78 (34.8%) were interrupted owing to toxic events. In particular, 21/78 (26.9%) cycles were interrupted because of grade III–IV hypotension, while 24/78 (30.1%) interruptions were determined by cardiac events (i.e. myocardial ischaemia; arrhythmias such as atrial fibrillation, supraventricular tachyarrhythmias, grade II atrial-ventricular block; myocarditis) (unpublished data). As NO production and release is involved either in vascular muscle dilation (Moncada *et al.*, 1991) or in direct myocardial damage (Paulus *et al.*, 1994), NO plasma concentration might correlate both with vascular and cardiac toxic events. Moreover, in a subset of 31 cycles in which echocardiography was routinely performed before and after each IL-2 infusion, a number of subclinical echocardiographic abnormalities were found, mainly regarding LV diastolic impairment, possibly suggesting a pathogenetic role of NO in damaging myocardial tissue (Di Lucca *et al.*, 1995). In the present study, plasma nitrate + nitrite levels correlate with hypotension, as already shown (Miles *et al.*, 1994), while no association was found between plasma nitrate + nitrite levels and the cardiac toxic events observed (two ischaemic ECG changes and one cardiogenic pulmonary oedema). This seems to suggest that NO is not the final mediator of myocardial damage where cardiac toxicity is concerned. Alternatively, the local release of NO via the inflammatory cascade of cytokines initiated by IL-2 might be enough to determine impairment of ventricular function, without a release in systemic circulation to levels detectable by plasma sampling. A more complex and invasive experimental design (e.g. intra right atrium blood sampling) would be needed to get full insight into this tissue.

In conclusion, the determination of plasma nitrate + nitrite levels during CIVI IL-2 can provide a useful estimate of the degree of peripheral vascular relaxation and capillary leakage associated with cytokine action, clinically manifested as hypotension. A dose-dependent pattern and course-dependent pattern for plasma nitrate + nitrite changes have been well established. However, isolated cardiac toxicity, which continues to represent a relevant problem during IL-2 therapy, does not appear to be predicted by plasma nitrate + nitrite changes. The mechanisms of this therapy-limiting toxicity require further studies, so that clinicians could reliably predict and adequately prevent cardiac events related to IL-2 infusions.

**Acknowledgement**

This work was partially supported by the CNR-Italy- Progetto Finalizzaro 'ACRO' No. 95.00437–PF39.

### References

BALCH CM, HOUGHTON AN AND PATERS LJ. (1993). Cutaneous melanoma. In *Cancer. Principles and Practice of Oncology*, De Vita VT Jr, Hellman S and Rosenberg SA. (eds) pp. 1612–1661. JB Lippincott: Philadelphia.

BESANA C, BORRI A, BUCCI E, CITTERIO G, DI LUCCA G, FORTIS C, MATTEUCCI P, TOGNELLA S, TRESOLDI M, BAIOCCHI C, LANDONIO G, GHISLANDI E AND RUGARLI C. (1994). Treatment of advanced renal cell cancer with sequential intravenous recombinant interleukin-2 and subcutaneous alpha-interferon. *Eur. J. Cancer*, **30A**, 1292–1298.

CIFONE MG, FESTUCCIA C, CIRONI L, CAVALLO G, CHESSA MA, PENSA V AND TUBARO E. (1994). Induction of the nitric oxide-synthesizing pathway in fresh and interleukin-2-cultured rat natural killer cells. *Cell Immunol.*, **157**, 181–194.

COX GW, MELILLO G, CHATTOPADHYAY U, MULLET D, FERTEL RH AND VARESIO L. (1992). Tumor necrosis factor-alpha-dependent production of reactive nitrogen intermediates mediates IFN-gamma plus IL-2-induced murine macrophage tumoricidal activity. *J. Immunol.*, **149**, 3290–3296.

DE BELDER AJ, RADOMSKY MW AND WHY HJF. (1993). Nitric oxide synthase activities in human myocardium. *Lancet*, **431**, 84–85.

DI LUCCA G, CITTERIO G, FRAGASSO G, ROSSETTI E, SCAGLIETTI U, TRESOLDI M, RUGARLI C AND CHIERCHIA SL. (1995). Assessment of interleukin-2-induced cardiovascular toxicity by echocardiography. *Eur. J. Cancer*, **31A**, S123.

FINKEL MS, ODDIS CV, JACOB TD, WATKINS SC, HATTLER BG AND SIMMONS RL. (1992). Negative inotropic effects of cytokines on the heart mediated by nitric oxide. *Science*, **257**, 387–389.

FOPPOLI M, CITTERIO G, POLASTRI D AND GUERRIERI R. (1995). The feasibility of repetitive courses of high-dose continuous intravenous infusion interleukin-2 and subcutaneous alpha-interferon with polichemotherapy in advanced malignant melanoma. *Tumori*, **81**, 102–106.

GREEN LC, WAGNER DA, GLOGOWSKI J, SKIPPER PL, WISHNOK JS AND TANNEBAUM SR. (1982). Analysis of nitrate, nitrite, and °15Nénitrate in biological fluids. *Ann. Biochem.*, **126**, 131–138.

HIBBS JB JR, WESTENFELDER C, TAINTOR R, VAVRIN Z, KABLITZ C, BARANOWSKI RL, WARD JH, MENLOVE RL, MCMURRAY MP AND KUSHNER JP. (1992). Evidence for cytokine-inducible nitric oxide synthesis from L-arginine in patients receiving interleukin-2 therapy. *J. Clin. Invest.*, **89**, 867–877.

KILBOURN RG, FONSECA GA, GRIFFITH OW, EWER M, PRICE K, STRIEGEL A, JONES E AND LOGOTHETIS CJ. (1995). NG-metyl-L-arginine, an inhibitor of nitric oxide synthase, reverses interleukin-2-induced hypothension. *Crit. Care Med.*, **23**, 1018–1024.

LINEHAN WM, SHIPLEY WU AND PARKINSON DR. (1993). Cancer of the kidney and ureter. In *Cancer. Principles and Practice of Oncology*, De Vita VT Jr, Hellman S and Rosenberg SA. (eds) pp. 1023–1042. JB Lippincott: Philadelphia.

MARGOLIN KA, RAYNER AA, HAWKINS MJ, ATKINS MB, DUTCHER JP, FISHER RI, WEISS GR, DOROSHOW JH, JAFFE HS AND ROPER M. (1989). Interleukin-2 and lymphokyne activated killer cell therapy of solid tumors: analysis of toxicity and management guidelines. *J. Clin. Oncol.*, **7**, 486–498.

MEYER J, LENTZ CW AND STOTHERT JC JR. (1994). Effects of nitric oxide synthesis inhibition in hyperdynamic endotoxemia. *Crit. Care Med.*, **22**, 306–312.

MILES D, THOMSEN L, BALKWILL F, THAVASU P AND MONCADA S. (1994). Association between biosynthesis of nitric oxide and changes in immunological and vascular parameters in patients treated with interleukin-2. *Eur. J. Clin. Invest.*, **24**, 287–290.

MONCADA S AND HIGGS A. (1993). The L-arginine-nitric oxide pathway. *N. Engl. J. Med.*, **329**, 2002–2012.

MONCADA S, PALMER RMJ AND HIGGS EA. (1991). Nitric oxide: physiology, pathophysiology, and pharmacology. *Pharmacol. Rev.*, **43**, 109–142.

OCHOA JB, CURTI B, PEITZMAN AB, SIMMINS RL, BILLIAR TR, HOFFMAN R, RAULT R, LONGO DL, URBA WJ AND OCHOA AC. (1992). Increased circulating nitrogen oxides after human tumor immunotherapy: correlation with toxic hemodynamic changes. *J. Natl Cancer Inst.*, **84**, 864–867.

OSANTO S, CLUITMANS FHM, FRANKS CR, BOSKER HA AND CLETON FJ. (1988). Myocardial injury after interleukin-2 therapy. *Lancet*, **2**, 48.

PAULUS WJ, VANTRIMPONT PJ AND SHAM AM. (1994). Acute effects of nitric oxide on left ventricular relaxation and diastolic distensibility in humans. Assessment by bicoronary sodium nitroprusside infusion. *Circulation*, **89**, 2070–2078.

PETROS A, LAMB G AND LEONE A. (1994). Effects of nitric oxide synthase inhibitor in humans with septic shock. *Cardiovasc. Res.*, **28**, 34–39.

RABER J AND BLOOM FE. (1994). IL-2 induces vasopressin release from the hypothalamus and the amygdala: role of nitric oxide-mediated signaling. *J. Neurosci.*, **14**, 6187–6195.

TAYLOR-ROBINSON AW, LIEW FY, SEVERN A, XU D, MCSORLEY SJ, GARSIDE P AND PADRON J. (1994). Regulation of the immune response by nitric oxide differentially produced by T helper type 1 and T helper type 2 cells. *Eur. J. Immunol.*, **24**, 980–984.

WANG P, BA ZF AND CHAUDRY IH. (1994). Nitric oxide. To block or enhance its production during sepsis? *Arch. Surg.*, **129**, 1137–1143.

WHITE RL, SCHWARTZENTRUBER DJ, GULERIA A, MACPHARLANE MP, WHITE DE, TUCKER E AND ROSENBERG SA. (1994). Cardiopulmonary toxicity of treatment with high dose interleukin-2 in 199 consecutive patients with metastatic melanoma or renal cell carcinoma. *Cancer*, **74**, 3212–3222.

WORLD HEALTH ORGANIZATION. (1979). *Handbook for Reporting Results of Cancer Treatment*. WHO Offset Publication No 48. WHO: Geneva.